# Supplementary material for: Mast cell extracellular granules are bioactive condensates assembled by heparin and polyamine
Source: Nat Chem Biol. 2026 Feb 27;22(7):1120–31. doi: 10.1038/s41589-026-02165-6 (PMC13010460; doi:10.1038/s41589-026-02165-6)
Supplement: Supplementary file 1 — Reporting Summary [file 41589_2026_2165_MOESM1_ESM.pdf]

nature portfolio

Corresponding author(s): Rohit V. Pappu and Xiaolei Su

Last updated by author(s): Jan 27, 2026

Reporting Summary

Nature Portfolio wishes to improve the reproducibility of the work that we publish. This form provides structure for consistency and transparency in reporting. For further information on Nature Portfolio policies, see our Editorial Policies and the Editorial Policy Checklist.

Statistics

For all statistical analyses, confirm that the following items are present in the figure legend, table legend, main text, or Methods section.

n/a	Confirmed	
☐	☒	The exact sample size (n) for each experimental group/condition, given as a discrete number and unit of measurement
☐	☒	A statement on whether measurements were taken from distinct samples or whether the same sample was measured repeatedly
☐	☒	The statistical test(s) used AND whether they are one- or two-sided<br>*Only common tests should be described solely by name; describe more complex techniques in the Methods section.*
☒	☐	A description of all covariates tested
☒	☐	A description of any assumptions or corrections, such as tests of normality and adjustment for multiple comparisons
☐	☒	A full description of the statistical parameters including central tendency (e.g. means) or other basic estimates (e.g. regression coefficient) AND variation (e.g. standard deviation) or associated estimates of uncertainty (e.g. confidence intervals)
☐	☒	For null hypothesis testing, the test statistic (e.g. F, t, r) with confidence intervals, effect sizes, degrees of freedom and P value noted<br>*Give P values as exact values whenever suitable.*
☒	☐	For Bayesian analysis, information on the choice of priors and Markov chain Monte Carlo settings
☒	☐	For hierarchical and complex designs, identification of the appropriate level for tests and full reporting of outcomes
☒	☐	Estimates of effect sizes (e.g. Cohen's d, Pearson's r), indicating how they were calculated

Our web collection on statistics for biologists contains articles on many of the points above.

Software and code

Policy information about availability of computer code

Data collection	Protein identification with Mass Spectrometry analysis was done using Q-Exactive Hybrid Quadrupole-Orbitrap Mass Spectrometer (LC-MS/MS) at the Protein Facility of the Iowa State University. Flow cytometry data was acquired using Cytek Spectroflo software (v. 3.3.0).
Data analysis	Mass Spectrometry raw data was analyzed using Thermo Scientific's Proteome Discoverer Software (v 3.1). MS and MS/MS data was searched using Mascot and Proteome Discoverer against publicly available databases on specific organism. Flow cytometry data was analyzed using Flowjo v10.10.0. Confocal images and Western blot images were analyzed using FIJI/ImageJ2 v2.16.0/1.54p.

For manuscripts utilizing custom algorithms or software that are central to the research but not yet described in published literature, software must be made available to editors and reviewers. We strongly encourage code deposition in a community repository (e.g. GitHub). See the Nature Portfolio guidelines for submitting code & software for further information.

Data

Policy information about availability of data

All manuscripts must include a data availability statement. This statement should provide the following information, where applicable:

- Accession codes, unique identifiers, or web links for publicly available datasets
- A description of any restrictions on data availability
- For clinical datasets or third party data, please ensure that the statement adheres to our policy

Data supporting the findings of this study are available in the main article and supporting information. Source data are provided with this paper.

Research involving human participants, their data, or biological material

Policy information about studies with human participants or human data. See also policy information about sex, gender (identity/presentation), and sexual orientation and race, ethnicity and racism.

Reporting on sex and gender	not applicable
Reporting on race, ethnicity, or other socially relevant groupings	not applicable
Population characteristics	not applicable
Recruitment	not applicable
Ethics oversight	not applicable

Note that full information on the approval of the study protocol must also be provided in the manuscript.

Field-specific reporting

Please select the one below that is the best fit for your research. If you are not sure, read the appropriate sections before making your selection.

☒ Life sciences ☐ Behavioural & social sciences ☐ Ecological, evolutionary & environmental sciences

For a reference copy of the document with all sections, see nature.com/documents/nr-reporting-summary-flat.pdf

Life sciences study design

All studies must disclose on these points even when the disclosure is negative.

Sample size	Sample sizes are indicated in the respective figures with circles/dots representing each replicates and the number of repeated experiments was stated in the figure legends. Samples sizes were based on common practices in the field (eg. Nat Chem Biol, 2025, 10.1038/s41589-025-01925-0).
Data exclusions	No data was excluded for the analysis in the study.
Replication	Biological or technical replicates were shown in the figures and disclosed in the respective figure legend.
Randomization	Randomization was performed during imaging for choosing imaging fields. Randomization for sample allocation is not performed because it is not relevant to the study. All samples received identical treatment and differences emerged from intrinsic biological variation.
Blinding	Blinding is not relevant to this study since no subjective analysis was used.

Reporting for specific materials, systems and methods

We require information from authors about some types of materials, experimental systems and methods used in many studies. Here, indicate whether each material, system or method listed is relevant to your study. If you are not sure if a list item applies to your research, read the appropriate section before selecting a response.

Materials & experimental systems

n/a	Involved in the study	
☐	☒	Antibodies
☐	☒	Eukaryotic cell lines
☒	☐	Palaeontology and archaeology
☐	☒	Animals and other organisms
☒	☐	Clinical data
☒	☐	Dual use research of concern
☒	☐	Plants

Methods

n/a	Involved in the study	
☒	☐	ChIP-seq
☐	☒	Flow cytometry
☒	☐	MRI-based neuroimaging

Antibodies

Antibodies used	1. anti-TNP IgE (clone TNP7, BioLegend #557079)<br>2. PE Cy7-FceR1a (Clone MAR-1, Biolegend #134318)

3. Alexa Fluor 488-CD117 (Clone 2B8, Biolegend #105816)
4. Alexa Fluor 647-ICAM-1 (Clone HA58, Biolegend #353114)
5. anti-spermine antibody (Abcam #ab26975)
6. Alexa Fluor 647 anti-rabbit IgG (H+L) (Invitrogen #A11008)
7. anti-TNFα (clone MP6-XT22, Biolegend #506302)
8. anti-IL-1β (clone 3A6, Cell Signaling Technology #4970)
9. anti-CPA3 (Proteintech #16236-1-AP)
10. anti-CD63 (clone NVG-2, Biolegend #143902)
11. anti-tryptase (clone 3G3, Bioss #bsm-52533R)
12. anti-b-actin (clone 13E5, Cell Signaling Technology #4970)
13. HRP conjugated goat anti-rat IgG (H+L) (Thermo Scientific # 31471)
14. HRP conjugated goat anti-rabbit IgG (H+L) (Thermo Scientific #31460)
15. HRP conjugated goat anti-mouse IgG (H+L) (Thermo Scientific # 31430)

Validation

All primary antibodies used in this study were validated by the respective manufacturer for the relevant applications. Detail validation statements for all antibodies can be accessed via the manufacturer's website catalog. Where applicable, antibody specificity was further confirmed either by the use of knockout cells/samples or appropriate isotype controls, or consistency with published studies.

Eukaryotic cell lines

Policy information about cell lines and Sex and Gender in Research

Cell line source(s)

HEK 293T (University of California, San Francisco, Cell Culture Facility)
Human umbilical vein endothelial cells (HUVEC; ATCC#PCS-100-013)

Authentication

The cell line vendors performed routine authentication. We will perform the STR profiling to get the most recent authentication by matching profiles of parental stocks.

Mycoplasma contamination

Verified for mycoplasma contamination, none detected.

Commonly misidentified lines
(See ICLAC register)

No misidentified cell lines have been used in this study.

Animals and other research organisms

Policy information about studies involving animals; ARRIVE guidelines recommended for reporting animal research, and Sex and Gender in Research

Laboratory animals

C57BL/6 and BALB/c mice (6-12 weeks)

Wild animals

No wild animals were used.

Reporting on sex

All mice used in this study were female.

Field-collected samples

No field-collected samples were used.

Ethics oversight

All Animal care and experiments were approved by the Yale University Institutional Animal Care and Use Committee

Note that full information on the approval of the study protocol must also be provided in the manuscript.

Plants

Seed stocks

not applicable

Novel plant genotypes

not applicable

Authentication

not applicable

Flow Cytometry

Plots

Confirm that:

☒ The axis labels state the marker and fluorochrome used (e.g. CD4-FITC).

☒ The axis scales are clearly visible. Include numbers along axes only for bottom left plot of group (a 'group' is an analysis of identical markers).

☒ All plots are contour plots with outliers or pseudocolor plots.

☒ A numerical value for number of cells or percentage (with statistics) is provided.

Methodology

Sample preparation	Primary cells were isolated as described in the methods. Cell surface staining was performed by incubation of cells with antibodies in PBS containing 2% BSA for 30 min at 4°C. Following antibodies were used: PE Cy7-FceR1a (Clone MAR-1, Biolegend), Alexa Fluor 488-CD117 (Clone 2B8, Biolegend), Alexa Fluor 647-ICAM-1 (Clone HA58, Biolegend). Isotype control was used to establish positive gate. For intracellular spermine staining, cells were fixed with 4% paraformaldehyde and then permeabilized with 0.5% saponin for 20 min. Cells were rinsed with PBS and incubated with anti-spermine antibody (Abcam, 1:500 dilution in PBS containing 2% BSA) overnight at 4°C, followed by incubation with Alexa Fluor 647 anti-rabbit secondary antibody (1:1000 dilution in PBS containing 2% BSA) for 1 h at room temperature. For MCEGs quantification, CountBright Absolute Counting Beads (Invitrogen) were spiked into MCEGs samples at Day 0. MCEGs were acquired under low-speed mode with a fixed volume, , then normalized to the counting bead number.
Instrument	Cytek Aurora
Software	SpectroFlo (v. 3.3.0) for data acquisition; Flowjo v10.10.0 for data analysis
Cell population abundance	Sorting is not used in this study. For analytic analysis, the population was determined by specific antibody staining and negative control (isotype controls and negative population).
Gating strategy	The gating strategy starts from the plot of FSC-H vs. SSC-A, live cells or small particles were gated out, followed by FSC-A vs. FSC-H gating to identify the singlets. The boundary of positive and negative population was drawn based on specific antibody staining and negative controls (isotype controls and negative population).

☐ Tick this box to confirm that a figure exemplifying the gating strategy is provided in the Supplementary Information.
