# Supplementary figures and images for: Mast cell extracellular granules are bioactive condensates assembled by heparin and polyamine
Source: Nat Chem Biol. 2026 Feb 27;22(7):1120–31. doi: 10.1038/s41589-026-02165-6 (PMC13010460; doi:10.1038/s41589-026-02165-6)

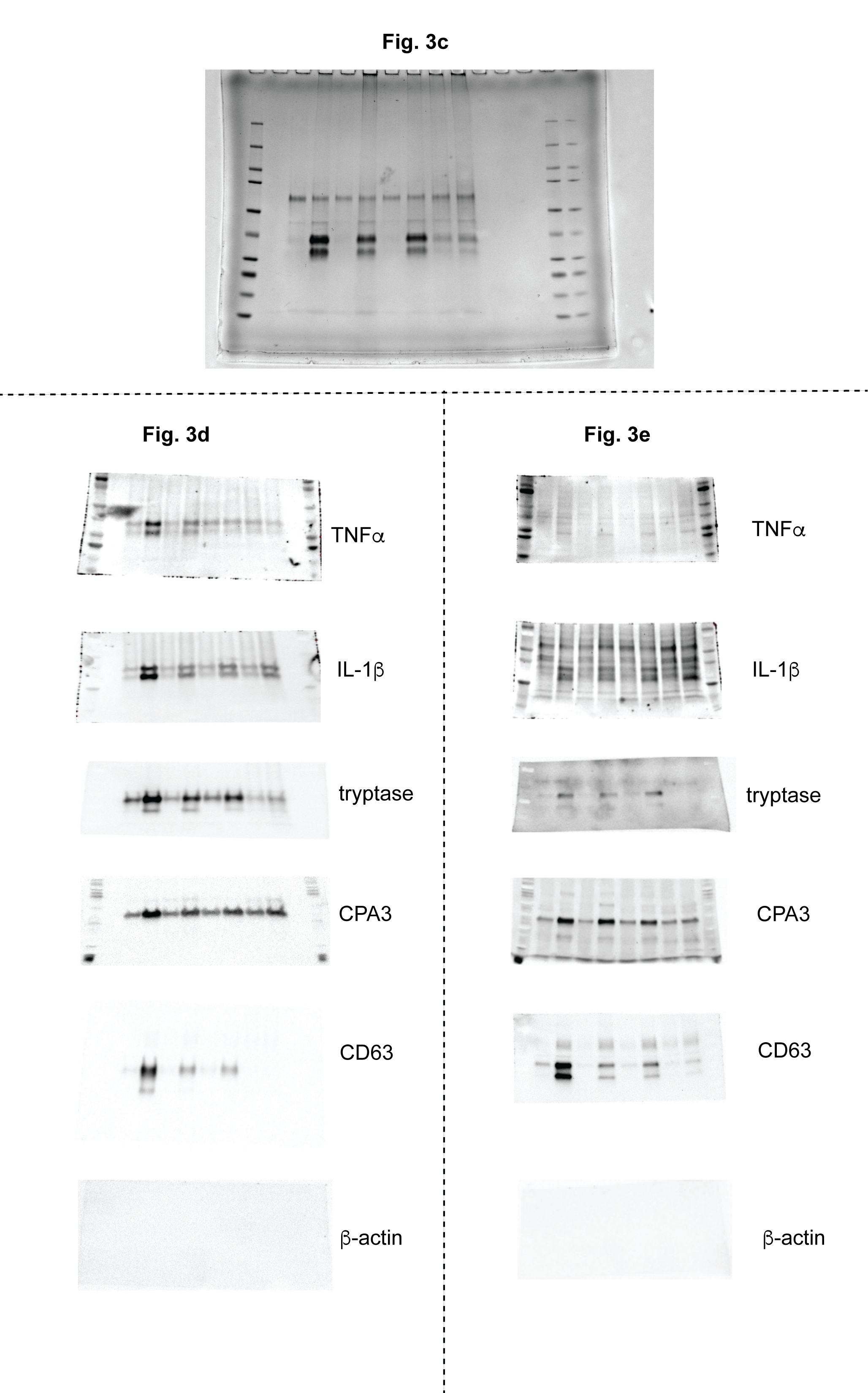

Supplement: Supplementary file 6 — Uncropped gel and blots. [file 41589_2026_2165_MOESM6_ESM.tif]

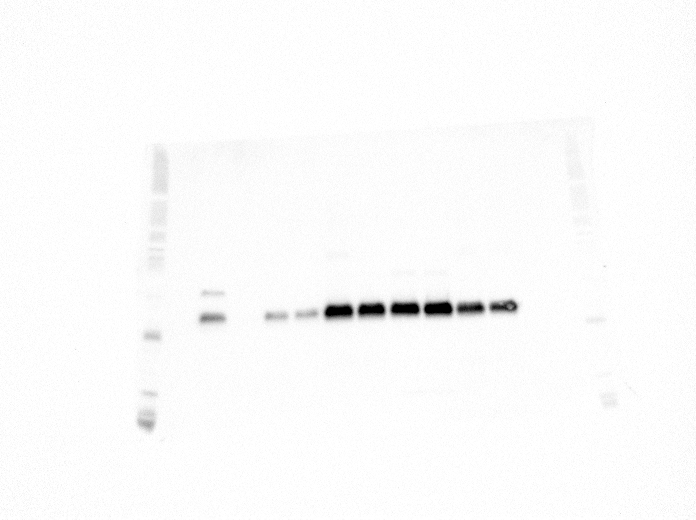

Supplement: Supplementary file 8 — Uncropped blot. [file 41589_2026_2165_MOESM8_ESM.tif]

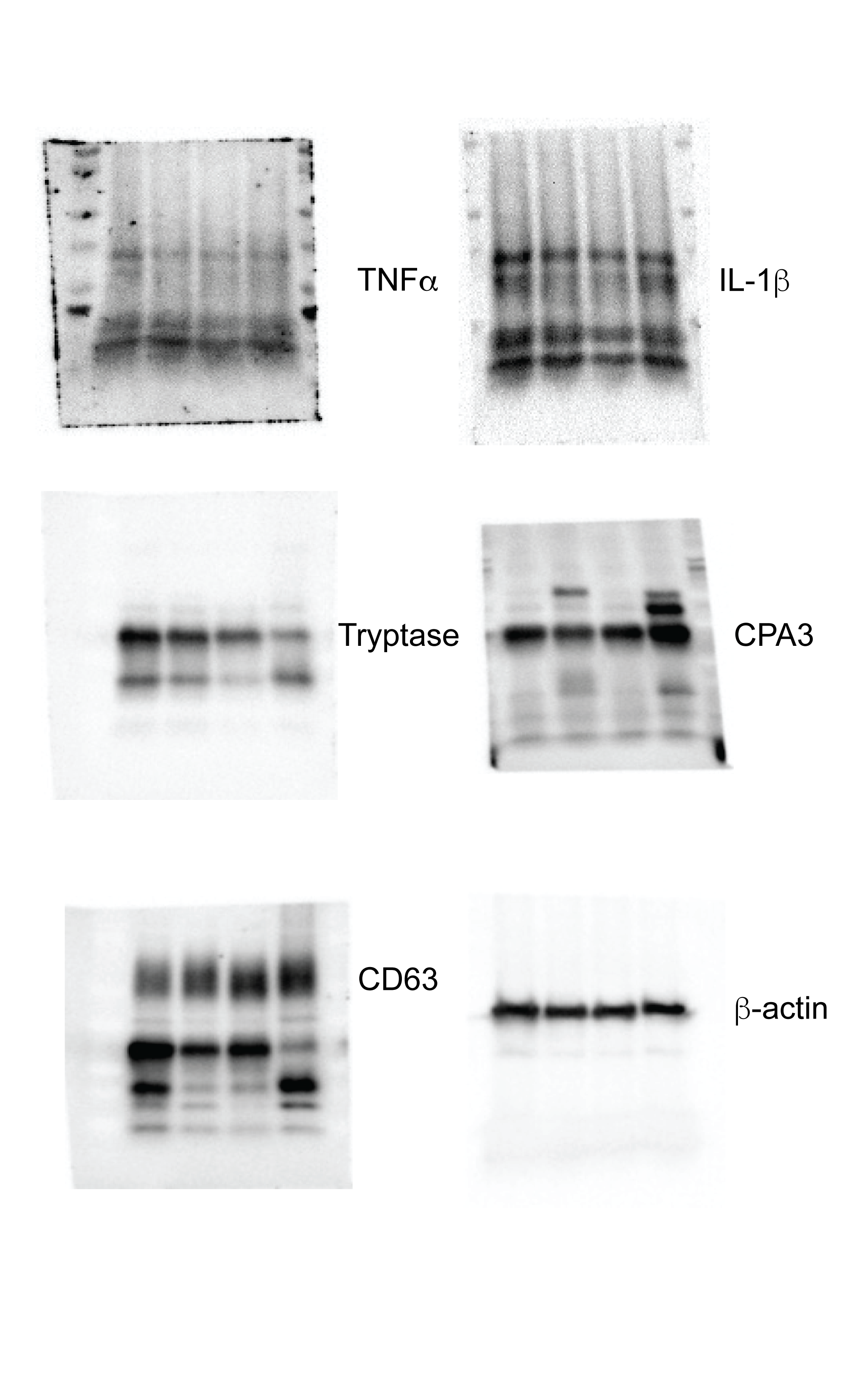

Supplement: Supplementary file 13 — Uncropped blots. [file 41589_2026_2165_MOESM13_ESM.tif]

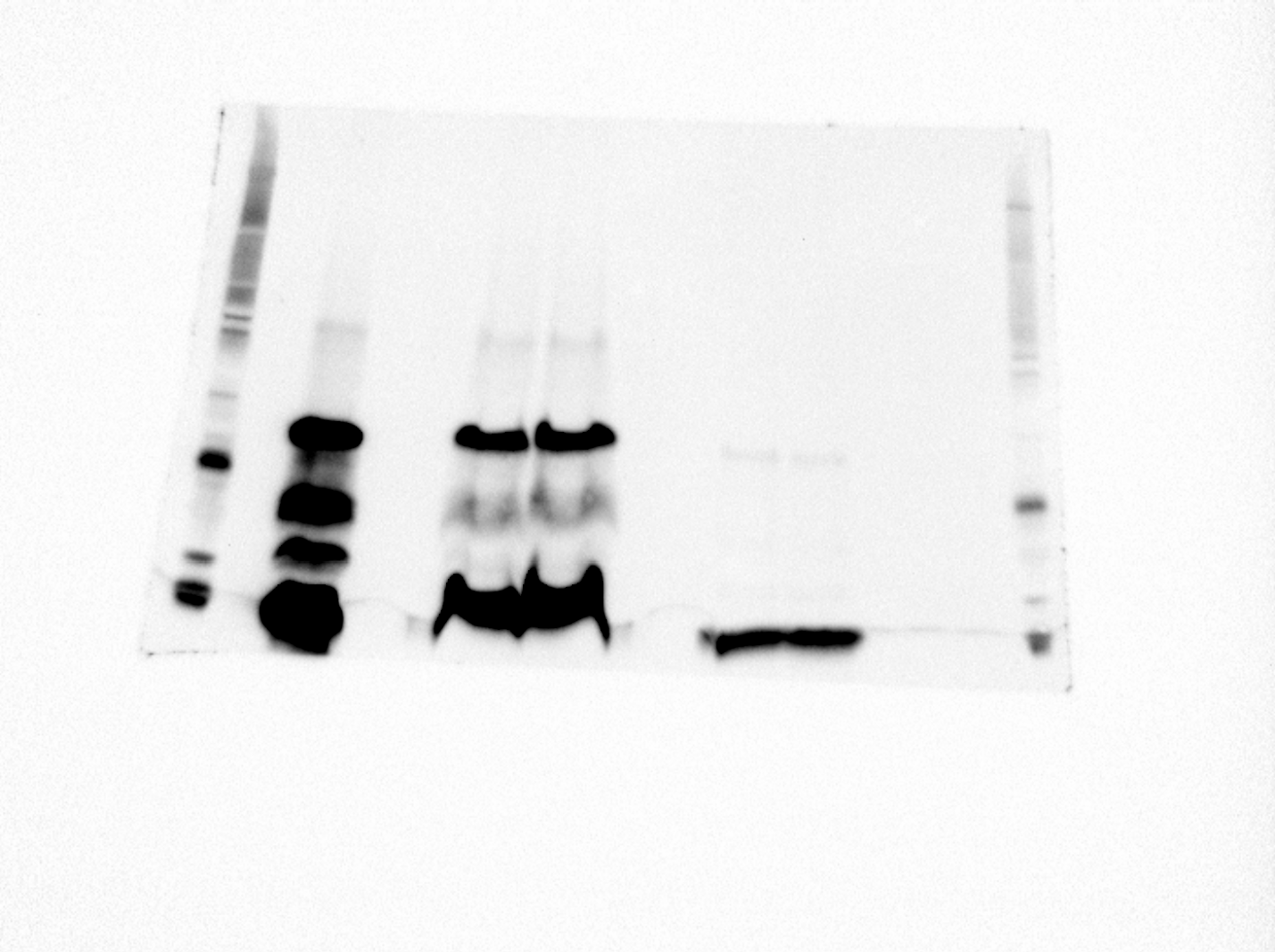

Supplement: Supplementary file 15 — Uncropped blot. [file 41589_2026_2165_MOESM15_ESM.tif]
